# Evaluation of pharmacotherapy of obstructive airway diseases in the Montenegrin outpatient care: comparison with two Scandinavian countries

**DOI:** 10.1186/2049-6958-7-12

**Published:** 2012-06-21

**Authors:** Natasha Duborija-Kovacevic, Milica Martinovic

**Affiliations:** 1 Department of Pharmacology and Clinical Pharmacology, Medical School of the University of Montenegro, Krusevac bb, 20000, Podgorica, Montenegro; 2 Department of Pathophysiology and Laboratory Medicine, Medical School of the University of Montenegro, Podgorica, Montenegro

**Keywords:** Obstructive airway diseases, Pharmacotherapy, Drug utilisation, Pharmacoepidemiology

## Abstract

**Background:**

This study is aimed at evaluating the pharmacotherapy of obstructive airway diseases (OAD) in the Montenegrin outpatient care (MOC) in 2010.

**Methods:**

Data on the reimbursed drugs which were prescribed during the reference period were obtained from the National Database that was established within the Health Insurance Fund of Montenegro in 2004. We have applied the standard pharmacoepidemiologic methodology with the defined daily dose (DDD) along with the Anatomical Therapeutic Chemical (ATC) classification of drugs. Clinical entities of OAD were classified according to the International Classification of Diseases (ICD-Revision X).

**Results:**

Prescribing and the subsequent use of drugs for OAD (ATC code R03) in 2010 was 18.18 DDD/1000inhabitants/day, much lower than in some developed countries. Fenoterol/ipratropium and salmeterol/fluticasone fixed combinations had the highest utilisation level, accounting for more than 50% of all OAD drugs. About 90% of OAD drugs were prescribed for COPD and asthma.

**Conclusions:**

Obtained results indicate that there are still large differences in OAD drug utilisation in MOC when compared with developed countries, but also some improvement in pharmacological approach to the pharmacotherapy of OAD in comparison to the earlier period.

## Background

Obstructive airway disease (OAD) is a respiratory disease category comprising several clinical entities. Although there are significant clinical, pharmacological, epidemiological, social and economic aspects typical of each of the entities, asthma and chronic obstructive pulmonary disease (COPD) are considered to have a particular impact on individuals, their families, health care system and the society in general.

Asthma is a worldwide problem, with the estimate of 300 million of affected individuals and global prevalence ranging from 1% to 18% of population across countries [1,2]. Medications for the asthma treatment can be classified as controllers or relievers. Controllers are medications taken on a daily and long-term basis to keep asthma under clinical control, mainly through their anti-inflammatory effect. Relievers are medications used on an as needed basis which act quickly to reverse bronchoconstriction and relieve its symptoms. Inhaled glucocorticosteroids are the most effective controller medications currently available. Rapid acting inhaled β_2_- agonists are the medications of choice for relief of bronchoconstriction and for the pretreatment of exercise-induced bronchoconstriction, in both in adults and children of all ages. Long acting inhaled β_2_-agonists (LABA) including formoterol and salmeterol, should not be used as monotherapy in asthma as these medication do not appear to influence the airway inflammation in asthma. They are the most effective when combined with inhaled glucocorticosteroids, and this combination therapy is the preferred treatment when a medium dose of inhaled glucocorticosteroids fails to achieve control of asthma. According to the Global Strategy for Asthma Management and Prevention, the goal of asthma treatment is to achieve and maintain clinical control [3].

COPD is the fourth largest cause of morbidity and mortality in the developed world [4] and it doubles the load on a healthcare system when it comes from elderly persons when compared to the age- and gender-matched controls [5]. Drugs treating COPD are exactly the same as those for asthma, except that long-acting anticholinergic drugs are more effective bronchodilators than LABA [6-9]. Bronchodilator medications are central to symptom management in COPD. Long- acting inhaled bronchodilators are convenient and more effective at producing maintained symptom relief than short-acting bronchodilators. Combining bronchodilators of different pharmacological classes may improve efficacy and decrease the risk of side effects compared to increasing the dose of a single bronchodilator. The efficacy and side effects of inhaled corticosteroids in asthma are dependent on the dose and type of corticosteroid, but whether this is also the case in COPD is unclear. Their role in the management of stable COPD is limited to specific indications. Long-term treatment with inhaled corticosteroids added to long-acting bronchodilators is recommended for patients at high risk of exacerbation [10]. In terms of the overall goals, the pharmacotherapy for COPD are directed at relieving symptoms, improving health status, limiting lung function decline, improving exercise tolerance, preventing deterioration, and decreasing mortality [3,11,12].

According to the Anatomical Therapeutic Chemical (ATC) classification of drugs [13], the drugs which are systemically or locally applied in the treatment of OAD have the R03 code. It divides drugs into several groups together with their combinations: selective and non-selective adrenoceptor agonists (R03A, R03C), glucocorticoids (R03BA), anticholinergics (R03BB), antiallergic agents (excluding corticosteroids) (R03BC), xanthines (R03DA), leukotriene receptor agonists (R03DC) and other newer drugs for OAD which cannot be classified within the previous groups (R03DX).

Unlike many developed countries, where the surveillance of drug utilisation has a rather long history and plays an important role in improving pharmacotherapy in many aspects, drug utilisation in Montenegrin outpatient care (MOC) according to DDD (defined daily dose)/ATC methodology was first investigated in 2000 [14]. Drugs for OAD were also subject to the above research. The findings highlighted the fact that the extent of prescribing these drugs was not optimal and needed improvement: the overall utilisation was much lower than in some developed countries, short-acting β_2_-agonists accounted for more than 50% and inhaled corticosteroids for only 13% of all prescribed drugs.

In order to evaluate the pharmacotherapy of OAD in MOC, the objective of this study was to analyse the utilisation of OAD drugs in 2010, comparing the findings with the available data on drug utilisation in developed countries, and analysing to what extent the OAD drugs were prescribed by individual OAD diagnoses, according to the International Classification of Diseases (ICD-Revision X).

## Methods

### Study design

This descriptive drug utilisation study comprises a sample out of 100% of reimbursed OAD drugs (ATC code R03) which were prescribed and subsequently used for the treatment of OAD in MOC in 2010.

### Data source

Introduction of the Information system named the *Control of the Distribution and Use of Drugs* into the Health Insurance Fund of Montenegro (HIFM) in 2004 and the implementation of electronic prescription in the Primary Health Care (PHC) in 2008, as part of the Drug Policy Reform Strategy, provided us with the 2010 data on the reimbursed drugs prescribed for the OAD treatment in the form of an electronic report. This report covered all drugs with ATC code R03 which were issued on prescription in state-owned pharmacies during the one-year period, including the generic and trade name of a drug, pharmaceutical form, the amount of pharmacologically active substance, diagnosis for which a medication was prescribed according to ICD-Revision X, the total number of DDD that were prescribed in 2010 for some diagnosis and costs in EUR. The number of DDD of a drug contained in the electronic report was converted into DDD/1000inhabitants/day (DTID), serving as a statistical measure of the outpatient drug use. According to the census from the beginning of 2011, the overall population of Montenegro in 2010 was approximately 620 000 [15].

### Reimbursement status of drugs for OAD in 2010

Drugs for OAD (ATC code R03) which were reimbursed by HIFM in 2010 are shown in Table 1.

**Table 1:** **Drugs for OAD which were reimbursed by HIFM (*Positive list*of OAD drugs) in 2010**

ATC code-4^th^ level Chemical/therapeutic subgroup	ATC code- 5^th^level	**Generic name**
R03AC	R03AC02	Salbutamol
Selective β2 adrenoceptor agonists	R03AC12	Salmeterol
R03AK	R03AK03	Fenoterol, ipratropium
Adrenergics and other drugs for OAD	R03AK06	Salmeterol, fluticasone
R03BA	R03BA01	Beclomethasone
Glucocorticoids	R03BA02	Budesonide
	R03BA05	Fluticasone
R03BC	R03BC01	Disodium
Antiallergic agents (excluding corticosteroids)		cromoglicate
R03DA	R03DA04	Theophylline
Xanthines	R03DA05	Aminophylline

Under the current legislation, only reimbursed drugs (so-called *Positive List of Drugs*) may be prescribed in PHC and issued on prescription in state-owned pharmacies without compensation. Other drugs, which are not on the Positive List, may be prescribed, but have to be paid for in state-owned or private pharmacies. It was found that about 30% of all the respiratory system drugs (ATC code R) in 2000 were purchased in private pharmacies [14]. Currently, there is no data available on the percentage of OAD drugs purchased in 2010, while the current Positive List has been almost twofold; this probably results in a decrease in the proportion of purchased drugs. Unfortunately, the application of the described methodology implied that those drugs could not be subject to this study.

### Utilization of drugs for OAD in MOC in comparison to developed countries

Norway and Finland were chosen for the evaluation of the obtained results against the accessible data from developed countries for several reasons. As developed Scandinavian economies with developed healthcare systems and the highest pharmacotherapeutic standards, these countries have a long history of drug utilisation surveillance and the accessible data on the different aspects of drug use.

### International Classification of Diseases (ICD), Revision X

The subject of this study was also based on the prescription of OAD drugs for ICD J42-J46 codes, including a number of clinical entities: unspecified chronic bronchitis-J42, emphysema-J43, chronic obstructive pulmonary disease-J44, asthma-J45, and status asthmaticus-J46. As already said herein, the 2010 data on prescribed drugs by specific diagnoses were collected from the electronic report.

## Results

The prescribing and subsequent use of drugs for OAD (ATC code R03) in MOC in 2010 is shown in Table 2.

**Table 2:** Prescribing of drugs for OAD (ATC code R03) in MOC in 2010, in DDD/1000 inhabitants/day (DTID) and in percent of total (%)

**Ord. No**	**ATC code**	**Generic name**	**Prescribing 2010** (**DTID)**	**Percent of total** (**%)**
1.	R03AK03	Fenoterol, ipratropium	6.11	33.6%
2.	R03AK06	Salmeterol, fluticasone	3.41	18.8%
3.	R03DA05	Aminophylline	2.43	13.4%
4.	R03BA05	Fluticasone	1.98	10.9%
5.	R03AC02	Salbutamol	1.89	10.4%
6.	R03AC12	Salmeterol	1.45	8.0%
7.	R03BA01	Beclomethasone	0.41	2.2%
8.	R03DA04	Theophylline	0.35	1.9%
9.	R03BA02	Budesonide	0.15	0.8%
10.	R03BC01	Disodium cromoglycate	0.00	0.0%
	R03	Total	18.18	100.0%

The overall use of drugs for OAD in MOC during the reference period was 18.18 DDD/1000inhabitants/day. The prescription of fixed combinations (fenoterol/ipratropium and salmeterol/fluticasone) accounted for the 52.4% share of all prescribed drugs. Aminophylline was ranked as the third, with 2.43 DTID (13.4%). Other drugs accounted for approximately one third of all prescribed drugs.

Table 3 provides a comparative overview of the utilization of drugs for OAD (ATC code R03) in MOC and in the two Scandinavian countries in 2010.

**Table 3:** Utilization of drugs for OAD in MOC in comparison to Norway and Finland in 2010, in DDD/1000 inhabitants/day (DTID) and in percent of total use

Ord. No	MOC 2010	Norway 2010	Finland 2009
	DTID (% of total use)	DTID (% of total use)	DTID (% of total use)
1.	Fenoterol, ipratropium 6.11 (33.6 %)	Salmeterol, corticosteroid 11.75 (18.4 %)	Formoterol and other drugs for OAD 10.86 (18.6 %)
2.	Salmeterol, fluticasone 3.41 (18.8 %)	Salbutamol 11.32 (17.7 %)	Salmeterol and other drugs for OAD 8.78 (15.0 %)
3.	Aminophylline 2.43 (13.4 %)	Ipratropium bromide 9.51 (14.9 %)	Salbutamol 8.10 (13.9 %)
4.	Fluticasone 1.98 (10.9 %)	Formoterol, corticosteroid 8.43 (13.2 %)	Fluticasone 5.49 (9.4 %)
5.	Salbutamol 1.89 (10.4 %)	Montelukast 4.70 (7.3 %)	Budesonide 5.39 (9.2 %)
6.	Salmeterol 1.45 (8.0 %)	Tiotropium bromide 4.36 (6.8 %)	Montelukast 5.16 (8.8 %)
7.	Beclomethasone 0.41 (2.2 %)	Fluticasone 3.11 (4.9 %)	Tiotropium bromide 3.50 (6.0 %)
8.	Theophylline 0.35 (1.9 %)	Terbutaline 2.94 (4.6 %)	Beclomethasone 3.27 (5.6 %)
9.	Budesonide 0.15 (0.8 %)	Budesonide 2.47 (3.9 %)	Terbutaline 2.05 (3.5 %)
10.	Othe 0.00 (0.0 %)	Other 5.38 (8.4 %)	Other 5.74 (9.8 %)
Total (R03)	18.18 (100.0 %)	63.97 (100.0 %)	58.34 (100.0 %)

As shown in Table 3, the total use of drugs for OAD in Norway in 2010 was 3.5 times the Montenegrin rate (63.97 vs. 18.18 DTID), while this difference against Finland was slightly smaller (58.34 vs. 18.18 DTID). The proportion of fixed combinations amounted to about 50% in MOC. In the observed Scandinavian countries, fixed combinations accounted for approximately one third (31.6% in Norway and 33.6% in Finland) of all the OAD drugs used. The most commonly used drug in MOC (fenoterol/ipratropium) made about one third of all prescribed drugs (6.11 DTID, 34%), but was used neither in Norway nor in Finland in 2010. On the other hand, drugs such as ipratropium bromide, montelukast, tiotropium bromide and formoterol in combination with corticosteroid were not prescribed in MOC.

The aim of this paper was also to analyse the extent to which OAD drugs (ATC code R03) were prescribed and used in MOC for individual OAD diagnoses, according to ICD-X Revision. The obtained results are provided in Table IV.

As shown in Table 4, 91% of OAD drugs were prescribed for COPD treatment (6.79 DTID, 49.7%) and asthma (5.64 DTID, 41.3%). Other clinical entities of OAD accounted for about 9% of the overall use.

**Table 4:** Prescribing of OAD drugs (ATC code R03) by diagnosis (ICD code J42-J46), in DDD/1000inhabitants/day (DTID) and percent (%) in 2010

**International Classification of Diseases (ICD)**	**Diagnosis**	**DTID**	**Percent of total use (%)**
J42	Unspecified chronic bronchitis	1.14	8.3
J43	Emphysema	0.05	0.4
J44	Other chronic obstructive pulmonary disease	6.79	49.7
J45	Asthma	5.64	41.3
J46	Status asthmaticus	0.04	0.3
	Total (J42-J46)	13.66	100.0

Given that OAD drugs were commonly used in 2010 to treat COPD and asthma, we also analysed their use against each of these clinical entities separately. Results are provided in Table 5 and Table 6.

**Table 5:** OAD drug use (ATC code R03) for COPD treatment (J44 according to ICD-X Revision) in 2010, in DDD/1000inhabitants/day (DTID) and percent (%)

**Ord. No**	**ATC code**	**Generic name**	**DTID**	**Percent of total use (%)**
1.	R03AK03	Fenoterol, ipratropium	2.62	38.6
2.	R03AK06	Salmeterol, fluticasone	1.20	17.7
3.	R03BA05	Fluticasone	0.85	12.5
4.	R03DA05	Aminophylline	0.81	11.9
5.	R03AC12	Salmeterol	0.75	11.1
6.	R03	Others	0.56	8.2
		Total	6.79	100.0

**Table 6:** OAD drug use (ATC code R03) for the treatment of asthma (J45 according to ICD-X Revision) in 2010, in DDD/1000inhabitants/day (DTID) and percent (%)

**Ord. No**	**ATC code**	**Generic name**	**DTID**	**Percent of total use (%)**
1.	R03AK06	Salmeterol, fluticasone	1.92	34.0
2.	R03AK03	Fenoterol, ipratropium	1.71	30.3
3.	R03BA05	Fluticasone	0.73	12.9
4.	R03AC02	Salbutamol	0.65	11.5
5.	R03DA05	Aminophylline	0.35	6.2
6.	R03	Others	0.28	5.1
		Total	5.64	100.0

The fixed combination of short-acting bronchodilators fenoterol and ipratropium was the most frequently used COPD treatment drug (39%, 2.62 DTID). Salmeterol/fluticasone accounted for approximately 18% (1.20 DTID) of the used, while fluticasone, aminophylline and salmeterol were used at almost equal rates (11-12%).

The fixed combination of salmeterol and fluticasone accounted for approximately one third of all drugs used for the asthma treatment in MOC in 2010 (34%, 1.92 DTID). Fenoterol/ipratropium was used slightly less (1.71 DTID, 30%) while other drugs (fluticasone, salbutamol, aminophylline) accounted for approximately one third of the overall use.

## Discussion

Since there is no accurate data on the prevalence of OAD in Montenegro, the obtained result of 18.18 DTID as a statistical measure of drug use cannot be used to draw any final conclusion on whether the use of OAD drugs, in terms of quantity, was optimal or not. In theory, if each patient took only one DDD of a single drug per day (which is often not the case), than the results obtained could indicate the 1.8% prevalence of OAD in the Montenegrin population.

The overall use of reimbursed OAD drugs in MOC increased by over 100% in 2010 when compared to 2000 [14]. It is certain that the obtained result does not match the increasing prevalence of OAD during the ten-year period, but it probably appears as a result of a number of influences, the individual contributions of which cannot be assessed by this study. The Positive List of Drugs was extended several times from 2000 to 2010, mostly due to a favourable socioeconomic situation in the country. As an illustration, gross domestic product (GDP) per capita in Montenegro increased by more than 2.5 times in 2009 comparing to 2000 [16]. An extended Positive List implies a wider choice for drug prescription by Montenegrin PHC physicians. Furthermore, new recommendations on the treatment of OAD at the global level, especially the introduction of fixed combinations, have contributed additionally to the obtained results.

Regarding the structure of drugs, we also observed some positive shift from the earlier period [14]. In 2000, bronchodilators were dominant, led by salbutamol and aminophylline, which could not be an indicator of rational pharmacotherapy. Under-use of controllers, particularly inhaled corticosteroids and prescription of non-asthma medications, such as antibiotics, expectorants and antitussives, were also observed in some developed countries during the same period [17-21]. In 2010, the fixed bronchodilator combination of fenoterol/ipratropium was the most frequently prescribed in MOC (34%). It could be subject to questioning because fenoterol was reported to cause disturbances in cardiac function and even increase the risk of death in patients with severe asthma, which is why some developed countries have removed this drug from the market [22-25]. A more frequent prescription of controllers such as inhaled corticosteroids, LABA and their combination in 2010 when compared to the earlier period, indicates that there was some improvement in the OAD pharmacological approaches. In addition, if apart from the structure of used drugs the corticosteroid/bronchodilator ratio is taken as a measure of quality of the OAD prescribing [26], a positive trend in the treatment of OAD within MOC can be noticed.

In comparison to Norway and Finland, the total OAD drug use in MOC is still of much smaller extent [27,28]. It could partially be explained by different population morbidity (Montenegro is a Mediterranean country) but also by different financial opportunities which influence the level of health care and legislation in relation to drugs. To illustrate the above, the World Bank database classifies the observed Scandinavian countries as ''high income'' ones, while Montenegro is an ''upper middle income'' country, with the 2010 GDP about 4.5 times lower than the one of Norway [29]. Furthermore, the Positive List of Drugs in MOC contained ten OAD drugs in 2010, while doctors in Norway and Finland had a much wider choice when prescribing. Still, it does not mean that a more rational prescribing practice could not be established within the existing MOC setting.

According to our results, the majority of OAD drugs used in 2010 were also prescribed for the treatment of COPD and asthma, as expected. The most commonly prescribed fixed combination of short-acting bronchodilators such as fenoterol and ipratropium could be partially justified when it comes to COPD, because bronchodilators are considered the first-line therapy in the management of this disease [11]. Nevertheless, it would be more appropriate if this was replaced with long-acting drugs, such as tiotropium bromide. However, since tiotropium bromide was not covered by the Positive List of Drugs in 2010, the physicians did not prescribe that drug. Or, to be more accurate, it probably was being prescribed by PHC physicians, but patients were buying it without reimbursement. The 18% proportion of the salmeterol/fluticasone prescription for the treatment of COPD can be considered as most likely rational. Many studies have found this combination to result in an improvement in FEV_1_ and disease-specific quality of life, and also to reduce the frequency of COPD deteriorations and hospitalizations [30-32].

According to the British Thoracic Society Guidelines for Asthma [33], the application of both inhaled corticosteroid and LABA is not recommended prior to introducing the third step (out of five) of asthma treatment. With fixed combinations, particularly the questionable combination of short-acting bronchodilators, accounting for about two thirds of all drugs for asthma treatment in 2010, the obtained results could indicate a possibly inadequate treatment of patients with mild and/or moderate form of disease which has been observed in some studies [34]. Additionally, the pharmacoeconomic aspect of OAD drug prescription is also very important [35,36] if we know that, according to HIFM data [37], the fenoterol/ipratropium combination accounted for approximately 22% of the overall costs for all drugs which act on the respiratory system (ATC code R) and about 2% of the total budget for drugs in 2009. To that extent, doctors need to be further informed and educated.

## Conclusions

In terms of both volume and type, there is still a much lesser extent of the OAD drug prescription in MOC in 2010 than in developed countries like Norway and Finland but it is remarkably larger when compared with the previous period. A larger share of controllers in the overall number of prescriptions could partly indicate a better pharmacotherapeutic approach to the OAD treatment. The frequent prescription of fixed combinations for COPD and asthma, especially of short-acting bronchodilators, requires additional analysis and possible implementation of a new regulation and/or educational activities among Montenegrin PHC prescribers. Furthermore, the observed positive trend should be encouraged in the future.

## Competing interests

The authors declare that they have no competing interests.

## Authors’ contribution

NDK has developed research, set goals and applied appropriate methodology. She wrote the first draft of the manuscript. MM participated in the introduction and discussion parts of the manuscript, and applied the relevant literature references. Both authors read and approved the final manuscript.
